# Pathological Calcium Signaling in Traumatic Brain Injury and Alzheimer’s Disease: From Acute Neuronal Injury to Chronic Neurodegeneration

**DOI:** 10.3390/ijms26189245

**Published:** 2025-09-22

**Authors:** Stephan Neuschmid, Carla Schallerer, Barbara E. Ehrlich, Declan McGuone

**Affiliations:** 1 School of Medicine and Health, Technical University of Munich, 81675 Munich, Germany; stephan.neuschmid@yale.edu (S.N.); carla.schallerer@yale.edu (C.S.); 2 Department of Pharmacology, Yale School of Medicine, New Haven, CT 06510, USA; 3 Department of Pathology, Yale School of Medicine, New Haven, CT 06510, USA

**Keywords:** axonal degeneration, beta-amyloid, calcium dysregulation, calpain, cognitive impairment, post-traumatic dementia, protein misfolding, tau, synaptic dysfunction, white matter injury

## Abstract

Loss of calcium homeostasis, a shared feature of Alzheimer’s Disease (AD) and Traumatic Brain Injury (TBI), activates enzyme-dependent cascades that promote protein misfolding, degrade synaptic architecture, impair axonal transport, and lead to neuronal death. Epidemiological studies identify TBI as a major risk factor for AD, yet the mechanistic basis for this association remains incompletely understood. Evidence from human and experimental studies implicate calcium dysregulation as a central link, triggering interconnected kinase, phosphatase, and protease networks that drive AD hallmark pathology, including amyloid-β (Aβ) accumulation and tau hyperphosphorylation. The calcium-dependent protease calpain is a key node in this network, regulating downstream enzyme activity, and cleaving essential scaffolding and signaling proteins. Selective vulnerability of the hippocampus and white matter to calcium-mediated damage may underlie cognitive deficits common to both conditions. In preclinical TBI and AD models, pharmacological inhibition of calcium-dependent enzymes confers neuroprotection. Recognizing disrupted calcium signaling as an upstream driver of post-traumatic neurodegeneration may enable early interventions to reduce AD risk among TBI survivors.

## 1. Introduction

Alzheimer’s Disease (AD), the leading cause of dementia, is projected to affect more than 150 million people globally by 2050 [1]. Age and genetic factors such as the ε4 allele of apolipoprotein E (*APOE4*) are established risks, but decades of epidemiological evidence has also identified Traumatic Brain Injury (TBI) as an important, potentially modifiable environmental factor for AD and all-cause dementia (ACD) [2]. Repetitive TBI accelerates cognitive decline, and a dose–response relationship exists between injury severity and risk of dementia [3]. Risk magnitude is influenced by age at injury, baseline cognitive reserve, *APOE4* genotype, and trauma biomechanics [4]. Epidemiological data indicate a significant association between TBI and dementia risk, whereas findings regarding AD are less robust, inconsistent, and show substantial statistical and clinical heterogeneity (Table 1). These discrepancies are partly attributable to differences in diagnostic criteria for TBI and AD, variations in study design, and incomplete reporting or characterization of trauma biomechanics, which may also introduce recall bias [5]. Differences in covariate adjustment and heterogeneity in the length of clinical follow-up (months to decades) limits conclusiveness and raises the possibility of reverse causation, for example, when dementia-related cognitive impairment increases the risk of falls and subsequent TBI. Despite inconsistent evidence for a direct causal link between TBI and AD, the high prevalence and frequent identification of TBI as a dementia risk factor warrants careful scrutiny and structured follow-up in clinical settings, particularly for repetitive or severe injuries. Because most TBI’s are mild and often self-limiting, and because TBI is highly heterogenous, the lifetime incidence of TBI-related neurodegeneration remains uncertain [6]. Current data are largely heterogenous and retrospective, limiting causal inference and underscoring the need for larger, well-characterized prospective cohorts and biomarker-based studies.

**Figure 1:**
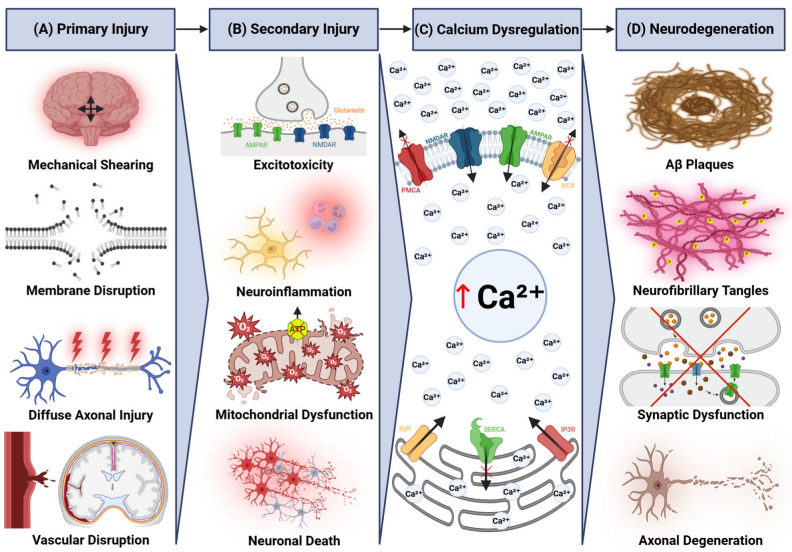
Schematic overview of TBI-induced neurodegeneration, highlighting calcium dysregulation as a central driver. (**A**) Primary injury mechanisms, including mechanical shearing, membrane disruption, diffuse axonal injury, and vascular disruption, cause initial damage that subsequently triggers (**B**) secondary injury cascades. These involve excitotoxicity, neuroinflammation, and mitochondrial dysfunction, promoting reactive oxygen species (ROS) production (labeled as O_2_^−)^, ATP depletion, neuronal death, and (**C**) disruption of calcium homeostasis. Elevated cytosolic calcium concentration (marked with red arrow) results from increased influx, reduced extrusion, and enhanced release from intracellular stores. (**D**) Sustained calcium imbalance activates chronic neurodegenerative pathways, resulting in Aβ and tau accumulation, synaptic dysfunction, and progressive axonal degeneration. Black arrows indicate physiological direction of transport, while red crosses denote dysfunction. Created with BioRender.com (https://www.biorender.com, accessed on 20 August 2025).

The strong epidemiologic and mechanistic associations between TBI and AD have focused attention on shared biological pathways linking acute neuronal injury to chronic neurodegeneration. Disruption of neuronal calcium homeostasis has emerged as a leading candidate mechanism [16,17]. Under physiological conditions, neurons maintain a steep calcium gradient with extracellular concentrations (~1 mM) approximately 10,000 times higher than cytosolic levels (~100 nM), which allows tightly regulated signaling for synaptic transmission, plasticity, gene transcription, and cell survival [18]. In TBI, this gradient collapses within seconds of injury due to excitotoxicity, mitochondrial and endoplasmic reticulum (ER) dysfunction, and membrane disruption [17]. In AD, calcium overload arises through mechanisms that include Aβ-induced calcium influx, tau-related organelle stress, and altered pump or channel function [19] (Figure 1). Loss of calcium regulation across neuronal subcellular compartments (e.g., the axon, synapse, and ER) activates overlapping kinase, phosphatase, and protease cascades which in turn promote Aβ and tau pathology [20]. Sustained intracellular calcium elevation is neurotoxic and consistently associated with neurodegeneration in human postmortem studies [21].

This review examines evidence linking calcium dysregulation after TBI to downstream neurodegenerative processes relevant to AD, with an emphasis on calcium-activated kinases (CDK5, GSK3β, PKC, CaMKII, ERK, and DYRK1A), phosphatases (PP2A and CaN), and proteases (calpains). Although these enzymes have been studied individually, their integrated network dynamics and contributions to vulnerability or resilience after TBI remain poorly defined [22]. Pharmacological inhibition of calcium-dependent enzymes reduces AD pathology in multiple preclinical models [23,24,25]. Collectively, these observations identify impaired calcium signaling as a central mechanistic link between TBI and chronic neurodegeneration.

## 2. Physiological Calcium Signaling and Enzymatic Regulation

Calcium, a ubiquitous second messenger, regulates synaptic transmission, plasticity, survival, and enzymatic activity. Its homeostasis depends on a highly coordinated network spanning distinct cellular compartments to ensure precise spatial and temporal control [26].

Following neurotransmitter binding or membrane depolarization, calcium enters the neuron through N-methyl-D-aspartate receptors (NMDARs), α-amino-3-hydroxy 5-methyl-4-isoxazolepropionate receptors (AMPARs), store-operated calcium (SOC) channels, transient receptor potential (TRP) channels, and voltage-gated calcium channels (VGCCs) [18]. In contrast, signal termination is mediated by sodium/calcium exchangers (NCXs) and the plasma membrane calcium-ATPase (PMCA), which extrude calcium to the extracellular space and prevent toxic accumulation [27].

In the ER, calcium modulates the inositol 1,4,5-trisphosphate receptor (IP3R) and ryanodine receptor (RyR) to promote further calcium release, a process known as calcium-induced calcium release (CICR) [28]. Restoration requires sarcoplasmic–endoplasmic reticulum calcium-ATPase (SERCA) pumps, which maintain the ER as the major calcium store [29].

Mitochondria buffer calcium using the mitochondrial calcium uniporter (MCU) and release it through mitochondrial NCX (mNCX), preventing overload and permeability transition [30]. Crosstalk between organelles occurs at mitochondria-associated ER membranes (MAMs), which coordinate calcium transfer from the ER to mitochondria [31]. Cytosolic buffering proteins (e.g., calmodulin, calbindin, parvalbumin, and S100) fine-tune signal amplitude and duration [32]. Together, this architecture generates spatially restricted calcium microdomains at presynaptic terminals, postsynaptic densities, and MAMs, enabling localized calcium transients without global calcium elevation [31,33].

Disruption of this network after TBI or in AD results in sustained calcium elevation, loss of microdomain compartmentalization, and aberrant activation of calcium-dependent enzymes [19] (Table 2). These enzymes include kinases, phosphatases, and proteases regulated by calcium or calcium-sensitive co-factors. Once activated, kinases phosphorylate targets, phosphatases counteract these modifications to maintain dynamic regulation, and proteases such as calpains cleave structural and signaling proteins [34,35]. Their combined activity shapes downstream signaling to determine neuronal fate.

## 3. Calcium Hypothesis

The calcium hypothesis of AD proposes that intracellular calcium dysregulation serves as an initiating driver of neurodegeneration, preceding and amplifying proteinopathies that define AD, including Aβ plaques and tau-containing neurofibrillary tangles (NFTs) [58]. Although age and genetic susceptibility (e.g., *APOE4*, presenilin mutations) remain major determinants of risk, converging experimental and human evidence demonstrates that TBI disrupts calcium homeostasis in ways that recapitulate early AD-like changes, providing a mechanistic link between acute injury and chronic degeneration [17,19]. Three core criteria support the calcium hypothesis: (1) calcium imbalance occurs prior to AD neuropathology and clinical symptoms; (2) disrupted calcium signaling acts as a convergent pathway for diverse AD risk factors, and (3) bidirectional interactions between calcium dysregulation and Aβ and tau pathology create a self-perpetuating cycle of injury and degeneration [59,60]. Genetic data support an upstream role for calcium dysregulation. Familial presenilin mutations increase ER calcium leak, leading to aberrant calcium release and neuronal hyperexcitability that precedes Aβ and tau pathology [61]. Despite this, the underlying molecular mechanisms remain incompletely defined [62], and no effective disease-modifying therapies have emerged [63]. This gap has renewed interest in calcium dysregulation as a central link between TBI and neurodegeneration.

In TBI, mechanical membrane disruption permits uncontrolled calcium influx [64], compounded by NCX reversal [65]. The resulting depolarization opens VGCCs and initiates excitotoxicity, where excess glutamate release drives further calcium entry through NMDAR and calcium-permeable AMPAR (CP-AMPAR), lacking the GluA2 subunit [66]. Metabotropic glutamate receptors (mGluRs) stimulate calcium release through IP3 signaling and calcium triggers CICR, progressively diminishing ER stores [67,68]. This depletion disrupts lysosomal calcium signaling by altering its pH and protein clearance, thereby aggravating AD-related proteinopathies [69]. Mitochondrial dysfunction and ATP shortage further compromise PMCA and SERCA activity, exacerbating calcium overload [70].

In AD, presenilin mutations and aging impair calcium buffering, receptor density, and organelle function [26]. These mutations sensitize IP3Rs [71], increase RyR expression [72], impair SERCA function [73], and alter ER leak channels [74], producing sustained calcium release and neuronal hyperexcitability. Presenilin mutant models develop neurodegeneration even in the absence of Aβ, supporting an upstream role for calcium dysregulation [75]. *APOE4* genotype further exacerbates calcium-driven synaptic loss, cognitive decline, and both tau and amyloid pathology following TBI and in AD [76,77].

Human genetic evidence further supports a calcium-centered mechanism by linking TBI and AD risk through shared polygenic architecture. A genome-wide association study (GWAS) from the VA Million Veteran Program (111,494 TBI cases) identified top signals including *NCAM1*, *APOE*, *FTO*, and *FOXP2*, with a single-nucleotide polymorphism (SNP)-based heritability of 0.060. Using bivariate mixer model (MiXeR) analysis, the authors demonstrated that AD harbored fewer risk variants (lower polygenicity), but each had stronger detectable effects (higher discoverability) compared to TBI, and that approximately 60% of influential AD variants are shared with TBI, despite near-zero genome-wide genetic correlation, implying mixed effect directions and shared architecture not fully captured by correlation analysis alone [78]. A separate GWAS, evaluating post-injury outcomes in the CENTER-TBI/TRACK-TBI cohort (5268 TBI cases), estimated a liability-scale heritability of 0.26 but found no genome-wide significant associations [79]. The authors attributed the null genome-wide findings to limited power and emphasized a need for larger, ancestrally diverse cohorts, harmonization of TBI phenotypes, and future replication studies. Although no canonical calcium-channel coding genes reached genome-wide significance in either GWAS, sub-threshold calcium-relevant signals in TBI and pathway-level findings in AD support calcium-related biology as a shared feature, supporting polygenic approaches that aggregate sub-threshold effects across calcium-regulatory genes, for example, using PGS-Depot or PGSFusion, to stratify TBI survivors by predicted risk of calcium-stress-related neurodegeneration [80].

Calcium imbalance drives ER stress, mitochondrial dysfunction, oxidative stress, impaired autophagy, lysosomal dysfunction, and neuroinflammation, all of which contribute to Aβ pathology [81,82,83,84]. Once Aβ accumulates, it further disrupts calcium homeostasis through several mechanisms, including membrane insertion to form calcium-permeable pores [85], NMDAR modulation (particularly NR2B subtypes) [86], and dysregulation of RyR and IP3R signaling [87,88]. NR2B upregulation correlates with hippocampal degeneration and cognitive decline [89], and pharmacological inhibition mitigates many of these effects [90].

Aβ oligomers induce oxidative stress [91] and mitochondrial injury [92] through NMDAR activation. L-type VGCCs mediate Aβ-induced calcium influx [93], and their inhibition is neuroprotective [94]. Aβ also promotes AMPAR internalization, impairing synaptic function [95], although selective AMPAR subtypes may exert neuroprotective effects by enhancing non-amyloidogenic (α-secretase-mediated) APP processing [96].

In both TBI and AD, calcium overload activates calpains, which cleave calcium-regulatory proteins and worsen dysregulation. Calpain degrades NCX and PMCA, impairing extrusion [97,98], and cleaves IP3R to enhance ER calcium release [99]. Additional substrates include VGCCs [100], AMPARs [101], and NMDARs [102], further perpetuating excitotoxicity and synaptic dysfunction. The centrality of these proteins is underscored by the fact that nearly all have been pharmacologically targeted in preclinical models, with several advancing to clinical trials and some incorporated into current FDA-approved therapeutic strategies [103].

## 4. From APP to Aβ: Calcium-Mediated Aβ Pathology

Following TBI, calcium dysregulation activates enzymatic cascades that promote amyloidogenic APP processing, resulting in early Aβ accumulation through mechanisms overlapping with core molecular events implicated AD [104]. Calcium-dependent processes triggered by TBI therefore contribute directly to AD-related neuropathology, establishing calcium imbalance as a plausible mechanistic link between acute neuronal injury and chronic neurodegeneration.

**Figure 2:**
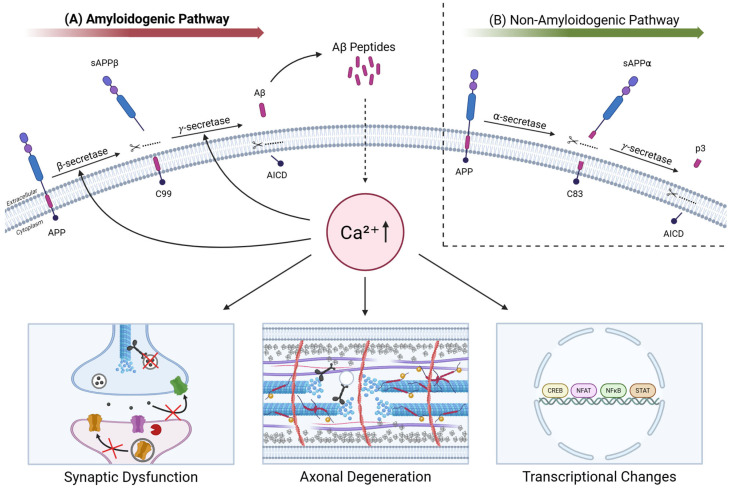
Integrative model of APP processing and Aβ-mediated calcium dysregulation driving downstream pathology. (**A**) In the amyloidogenic pathway, β-secretase generates a soluble N-terminal (sAPPβ) and C-terminal fragment (C99), which γ-secretase processes into pathological Aβ peptides and APP intracellular domain (AICD). Aβ accumulation in turn triggers calcium dysregulation, activating enzymatic cascades that cleave or aberrantly phosphorylate structural and signaling proteins. These changes impair vesicle trafficking and recycling, reduce postsynaptic receptor density, destabilize axons and synapses, and alter transcriptional regulation—ultimately promoting synaptic dysfunction, axonal degeneration, and persistent transcriptional changes. Additionally, Aβ-induced calcium dysregulation further enhances amyloidogenic processing, establishing a self-perpetuating cycle. Black arrows depict a simplified representation of pathological calcium signaling and downstream pathologies triggered by Aβ. (**B**) In the non-amyloidogenic pathway, α-secretase cleaves APP within the Aβ domain to generate sAPPα and C83. Subsequent γ-secretase cleavage yields p3 peptide and AICD. Created with BioRender.com (https://www.biorender.com, accessed on 20 August 2025).

APP, a type I transmembrane glycoprotein enriched in neurons, can undergo two mutually exclusive proteolytic pathways (Figure 2). In the non-amyloidogenic pathway, α-secretase (primarily ADAM10) cleaves APP within the Aβ domain, precluding Aβ formation [105]. In contrast, amyloidogenic processing begins with β-secretase (BACE1) cleavage, generating the C-terminal fragment C99, which is subsequently cleaved by γ-secretase (with presenilin as a catalytic subunit), predominantly producing Aβ40 and Aβ42 [106]. Aβ42 is particularly prone to aggregation and neurotoxicity [107].

TBI induces rapid APP upregulation and Aβ accumulation, particularly Aβ42, in the brain and cerebrospinal fluid (CSF) within hours of injury [108,109,110]. Animal models demonstrate that TBI-induced Aβ oligomers share conformational features with those in AD [111], and are associated with hippocampal atrophy and cognitive impairment [112]. Experimental TBI accelerates intra-axonal Aβ accumulation, lipid peroxidation, tau pathology, and cognitive impairment in transgenic models [113,114]. Both BACE1 expression and γ-secretase components, including presenilin-1, are upregulated in the hippocampus and cortex within 24–72 h of injury [115,116,117], and their inhibition reduces Aβ accumulation and cognitive decline [118].

Elevated intracellular calcium influences multiple steps of the amyloidogenic cascade. Calcium enhances acidification of endosomal and lysosomal compartments, optimizing BACE1 catalytic environment [119,120,121], and calcium/CaM binding further increases BACE1 enzymatic activity by approximately 2.5-fold in vitro [122].

The calcium-dependent protease calpain acts as a central mediator of post-traumatic neurodegenerative signaling. Normally restrained and tightly regulated by the endogenous inhibitor calpastatin [123], calpain becomes activated within minutes after TBI, persisting for days to weeks [56]. In AD, calpain is similarly elevated [124], where it promotes Aβ pathology by upregulating BACE1 expression [125]. Inhibition of calpain reduces BACE1 activity, Aβ accumulation, neuroinflammation, and memory deficits [25,125].

Following both controlled cortical impact [126] and blast exposure [127], calpain cleaves the CDK5 activator p35 to p25, resulting in sustained CDK5 activation and altered substrate specificity [128]. CDK5/p25 phosphorylates APP at crucial site Thr668, thereby enhancing its trafficking and susceptibility to BACE1 cleavage [129,130]. It also phosphorylates BACE1 directly, and the transcription factor STAT3, which in turn further upregulates BACE1 and presenilin-1 expression [131,132,133]. These effects are reversible with CDK5 inhibition and have been observed in transgenic models and human AD brains [23,131].

GSK3β activity, enhanced by p25 binding [134] and calpain-mediated cleavage [135], further drives amyloidogenesis. GSK3β phosphorylates APP at Thr668, and BACE1 at Thr252, promoting Aβ accumulation [136,137], whereas inhibition of GSK3β reduces Aβ hallmark pathology [138]. GSK3β activity is increased in AD brains [139] and strengthened by familial AD presenilin-1 mutations [140,141]. After TBI, GSK3β is transiently inhibited by protein kinase B (PKB)-mediated phosphorylation [142], but becomes persistently activated in the subacute phase, contributing to secondary injury [143].

PKC activity is dynamically elevated post-TBI [144], and has been identified as an early and persistent kinase targeting multiple AD-associated core proteins [145]. Calcium directly activates conventional PKC isoforms [48], whereas others undergo calpain-mediated cleavage to alter activity [146]. PKCε promotes non-amyloidogenic α-secretase cleavage, but is reduced in AD, possibly due to inhibition by GSK3β or Aβ peptides [147,148]. In contrast, PKCδ, activated in ischemia and AD [149], correlates with BACE1 expression, and enhances Aβ production. Inhibition or knockout of PKCδ reduces Aβ pathology [150]. Other PKC isoforms such as PKC-λ/ι promote BACE1 transcription by phosphorylation of NF-κB [151]. CaMKII may also contribute by phosphorylating APP at Thr654/Ser655 and activating NF-κB, although in vivo evidence remains limited [152,153].

Phosphatases oppose amyloidogenic processing. PP2A dephosphorylates APP at Thr668, suppressing Aβ production [154], but its activity is diminished in TBI and AD, leading to sustained APP phosphorylation and BACE1-mediated cleavage [155,156]. Aβ inhibits PP2A, amplifying cell injury [157]. PP2A reactivation attenuates amyloidogenic processing by modulating BACE1 and presenilin [158], while also reducing both astrogliosis and expression of the senescence marker p21 [159]. CaN, activated by calcium/CaM binding or calpain-mediated cleavage [160], enhances BACE1 expression through NFAT signaling promoting excitotoxicity and neuroinflammation, whereas inhibition reduces Aβ pathology and cognitive decline [161,162,163].

MAPK/ERK, activated downstream of calcium and calpain [164], increases BACE1 activity through DRP1 and STAT1 phosphorylation [165,166]. DRP1 is also phosphorylated by CDK5 and GSK3β, and dephosphorylated by PP2A and CaN, thereby linking aberrant mitochondrial fission with increased neuronal vulnerability to Aβ-induced toxicity [167]. Aβ oligomers further activate ERK, establishing a self-amplifying cycle [168]. DYRK1A phosphorylates APP and presenilin, leading to increased enzymatic activity and Aβ accumulation [169,170]. Calcium overload also promotes ER calcium leak, mitochondrial uptake, and unfolded protein response (UPR) activation, which upregulates BACE1 through eukaryotic initiation factor 2α (eIF2α) [171,172].

Together, these pathways define a calcium-APP-Aβ axis activated by TBI and sustained in AD. Central mediators include calpain, CDK5, GSK3β, PKC isoforms, DYRK1A, MAPK/ERK, PP2A, and CaN, all of which influence BACE1 or γ-secretase activity through transcriptional or posttranslational modifications. Their activation by calcium dysregulation supports the view that calcium imbalance is not a consequence, but rather a proximal driver of Aβ pathology in both acute injury and chronic neurodegeneration.

## 5. Tau Pathology: Calcium-Dependent Disruption of Microtubule Homeostasis

**Figure 3:**
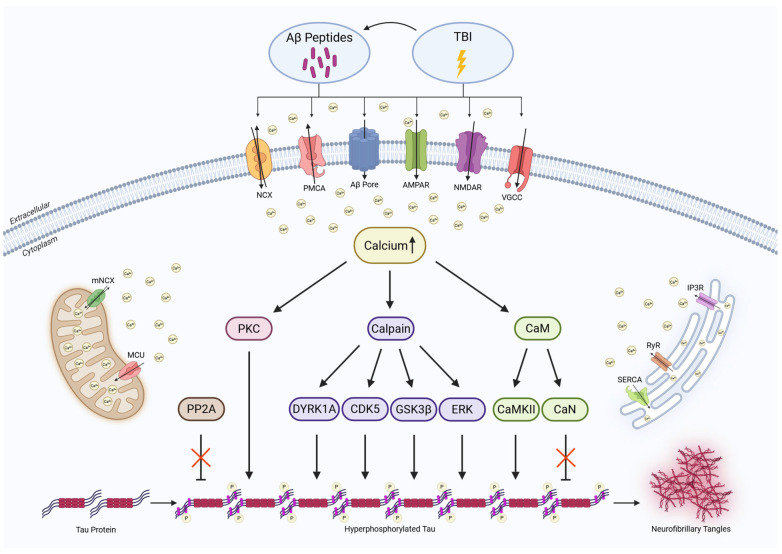
TBI- and Aβ-induced calcium dysregulation activates calcium-dependent signaling cascades that promote tau hyperphosphorylation and NFT formation. Excess calcium influx from the extracellular space and increased release from intracellular stores (ER and mitochondria) cause sustained cytosolic calcium elevation. This overload activates kinases (PKC, DYRK1A, CDK5, GSK3β, ERK, CaMKII), proteases (calpains), and co-factors (CaM), while impairing phosphatases (PP2A, CaN), thereby shifting the balance towards phosphorylation. This enzymatic imbalance promotes tau hyperphosphorylation, detachment, misfolding, aggregation, and ultimately NFT formation. Black arrows indicate a potential activation pattern of the interconnected enzyme network triggered by calcium dysregulation, whereas red crosses denote loss of physiological dephosphorylation capacity. Labeled calcium-signaling proteins mediate cytosolic calcium overload, with black arrows indicating the physiological direction of calcium transport. Created with BioRender.com (https://www.biorender.com, accessed on 20 August 2025).

Hyperphosphorylated tau accumulation is a defining feature of AD and other tauopathies including chronic traumatic encephalopathy (CTE), which is associated with repetitive mild TBI [173]. Tau, encoded by the *MAPT* gene on chromosome 17, exists as six isoforms, generated by alternative splicing [174]. Under physiological conditions, tau stabilizes microtubules and supports axonal transport through interactions with dynein and kinesin motor proteins. These functions are regulated by posttranslational modifications, particularly phosphorylation at multiple serine and threonine residues [175]. Under pathological conditions, tau becomes hyperphosphorylated, detaches from microtubules, and aggregates into β-sheet-rich paired helical filaments (PHFs) that form intracellular NFTs [176]. This process is driven by an imbalance between kinases and phosphatases, many of which are calcium-sensitive. Disruption of calcium homeostasis after TBI promotes tau phosphorylation and aggregation, linking acute injury to chronic tau pathology [177] (Figure 3).

Calcium dysregulation and tau pathology reinforce each other in a self-perpetuating cycle. Hyperphosphorylated tau reduces nuclear calcium levels and perturbs ER and mitochondrial calcium handling and communication, while suppressing CREB-mediated transcription, increasing neuronal vulnerability [178,179]. Age-related loss of the calcium-buffering protein calbindin correlates with increased NFT formations [180]. RyR-mediated calcium leak promotes tau hyperphosphorylation [181], whereas, in vitro, tau impairs mitochondrial calcium extrusion and promotes overload by mNCX inhibition, causing caspase-mediated death [182]. Pathological tau can also induce aberrant calcium influx, generating spontaneous calcium oscillations [182].

Experimental TBI models demonstrate early tau pathology, with abnormal phosphorylation detectable within 24 h and persisting for days [113]. Postmortem human studies confirm elevated levels of hyperphosphorylated tau after TBI [117]. Although NFTs are typically absent in acute injury [110], they can develop years later in survivors [183,184]. Even a single moderate TBI increases long-term risk of chronic tauopathy [185]. Beyond phosphorylation, tau acetylation promotes mislocalization and aggregation, with overlapping modifications observed in TBI and AD [186].

### 5.1. Kinase Hyperactivity

Multiple calcium-sensitive kinases implicated in AD are activated after TBI, where they conspire to drive tau hyperphosphorylation and NFT formation through reciprocal priming and feed-forward amplification.

**GSK3β functions as a central effector:** GSK3β phosphorylates more than 40 serine/threonine residues on tau, destabilizing microtubules and promoting detachment [20]. Pathological tau in turn alters GSK3β acetylation to prevent degradation, creating a feed-forward loop [187]. Calpain cleavage removes GSK3β’s autoinhibitory N-terminal domain, generating truncated forms with increased activity [188]. These fragments correlate with calpain activation and tau phosphorylation in AD and injury models [189], and also interact with PP2A, promoting GSK3β dephosphorylation and further enhancing its activity [190]. Presenilin-1 binds both tau and GSK3β, facilitating spatial proximity and linking tau phosphorylation with amyloidogenic APP processing [140]. Pharmacological inhibition (e.g., lithium) reduces tau phosphorylation and improves cognition in TBI models [191].

**CDK5 acts as a priming kinase:** CDK5 phosphorylates tau at residues that enhance subsequent GSK3β-mediated phosphorylation, accelerating and amplifying hyperphosphorylation [192]. Apart from direct tau phosphorylation [193], CDK5/p25 modifies hundreds of other substrates involved in calcium signaling and neurodegeneration, thereby exacerbating NFT formation and calcium dysregulation [194]. Elevated p25 levels are associated with early-onset AD pathology [195], and CDK5 inhibition confers neuroprotection in rotational TBI models [194].

**CaMKII propagates tau pathology:** Direct calcium/CaM binding induces CaMKII autophosphorylation at Thr286, increasing activity and driving redistribution from the cytosol to membranes within 30 min of TBI [196]. CaMKII phosphorylates tau at multiple sites [197], and promotes additional calcium influx by phosphorylating CP-AMPARs [198]. Calpain-generated CaMKII fragments display constitutive activity [199], priming tau for further phosphorylation by GSK3β and CDK5 [200]. Persistent CaMKII activation impairs synaptic plasticity and memory consolidation in transgenic mice, compounding post-injury cognitive decline [201].

**DYRK1A and ERK serve as auxiliary kinases:** DYRK1A phosphorylates tau [202], potentially priming it for subsequent GSK3β phosphorylation [203]. This cascade is amplified by calpain-mediated cleavage, which generates hyperactive DYRK1A fragments that are elevated in human AD brains and CSF [204]. ERK similarly phosphorylates tau at multiple sites [205] and is upregulated early in AD, correlating with NFT progression [206]. Both kinases promote neuroinflammation by activating cytokine pathways. Pharmacological inhibition with calcium channel blockers (e.g., lomerizine) reduces tau pathology and dampens proinflammatory responses, likely by indirectly modulating GSK3β and DYRK1A activity, although lomerizine is not a selective DYRK1A inhibitor [207].

**PKC modulates context-dependent effects:** Because certain isoforms have been implicated in both neuroprotection and neurotoxicity, PKC’s overall role remains context-dependent. Some isoforms phosphorylate tau [48], whereas others inhibit GSK3β, thereby promoting sensorimotor recovery and structural remodeling after TBI [208]. PKC activation reduces tau phosphorylation in experimental models, suggesting that targeted modulation may offer therapeutic benefit [209].

### 5.2. Phosphatase Failure

In AD, phosphatase activity is markedly reduced, particularly PP2A, which shows decreased expression, inhibition, and altered regulation, thereby failing to counterbalance kinase hyperactivity and shifting the balance towards pathological tau phosphorylation [210].

PP2A is responsible for approximately 70% of tau dephosphorylation in the human brain [211]. Both TBI and AD exhibit reduced PP2A activity, leading to sustained tau hyperphosphorylation [212]. Injury severity correlates with tau phosphorylation levels and with increased GSK3β and decreased PP2A expression, highlighting their inverse regulation [213]. Pharmacological activation of PP2A (e.g., sodium selenate) reduces tau phosphorylation and improves cognition in both TBI and AD models [212,214]. PP2A also suppresses ERK signaling; therefore, its loss amplifies tau pathology across multiple kinase pathways [215]. Calpain-mediated cleavage of PP2A’s α4 regulatory subunit directly impairs its assembly and function [216]. Additionally, acidic pH, in injured or degenerating tissue, activates asparaginyl endopeptidase (AEP), which cleaves the endogenous PP2A inhibitor SET/I2^PP2A^. The truncated inhibitor translocates to the cytosol, where it further suppresses PP2A activity, and promotes tau hyperphosphorylation and amyloidogenic processing [217,218].

CaN plays a paradoxical role in tau phosphorylation. Although it can directly dephosphorylate tau, calpain-mediated truncation removes its autoinhibitory domain, producing constitutively active fragments [219]. These fragments indirectly result in tau phosphorylation, potentially by modulating GSK3β activity [24]. Truncated CaN correlates with NFT density in AD brains [160], and remains persistently elevated for weeks after TBI, particularly in hippocampal and cortical regions [220]. The impact of CaN inhibition is context-dependent and in some models, it paradoxically exacerbates tau phosphorylation [221]. CaN also contributes to synaptic and neuronal loss by dephosphorylating the pro-apoptotic protein Bcl-2-associated death promoter (BAD), enabling its mitochondrial translocation and initiation of programmed cell death [222].

### 5.3. Proteolytic Cleavage

In addition to phosphorylation, tau undergoes proteolytic cleavage, generating fragments with increased aggregation potential and toxicity. Calpain and caspases, both activated downstream of calcium dysregulation, are central mediators of this process.

Calpain cleaves tau to generate neuron-specific N224 and 17-kDa fragments. N224, detectable in CSF, has been proposed as a candidate AD biomarker, because its levels correlate with cognitive decline [223,224]. The neurotoxic 17-kDa fragment damages the cytoskeleton, perturbs axonal transport, and contributes to synaptic dysfunction [225]. Calpain also disrupts mitochondrial dynamics by cleaving DRP1 and triggers the UPR, thereby amplifying neuronal stress [226,227]. In experimental TBI, calpain activity scales with cell death, and pharmacological inhibition blocks tau phosphorylation, delays NFT formation, reduces lesion volume, and preserves axonal integrity [228,229].

Caspases, activated by Aβ and potentially calpain, cleave tau at Asp421, producing truncated forms with high aggregation propensity that localize to early NFTs [230,231]. These fragments correlate with disease progression [232], and drive both tau-dependent [233] and tau-independent [234] cell death pathways.

Proteolytic cleavage acts at multiple points both upstream and downstream in the pathological cascade of tau phosphorylation. These interactions, driven by calcium overload, enzyme activation, fragment production, mitochondrial dysfunction, and cytoskeletal collapse, link acute injury to chronic propagation of tau pathology.

## 6. Synaptic Dysfunction

Synaptic loss is one of the strongest pathological correlates of cognitive decline in TBI and AD, often preceding overt hallmarks such as amyloid plaques or NFTs [235]. Because synaptic function, particularly long-term potentiation (LTP), depends on tightly regulated spatiotemporal calcium signaling, synapses are highly vulnerable to calcium imbalance [33]. Under physiological conditions, NMDAR-mediated calcium influx activates CaMKII, which promotes insertion of GluA1-containing AMPAR subunits into the postsynaptic membrane, strengthening synaptic efficacy [236].

In TBI and AD, sustained calcium dysregulation drives persistent calpain activation, which cleaves GluA1, NMDAR subunits, and scaffolding proteins such as PSD-95, disrupting LTP [237,238]. Calpain also degrades cytoskeletal components (spectrin, tubulin, MAPs, etc.), compromising synaptic integrity and vesicle trafficking [57]. Cleavage of presynaptic protein GAP43 impairs neuronal plasticity [239], and processing of dynamin-1 disrupts synaptic vesicle recycling and memory function [240]. These effects are compounded by CDK5, DYRK1A, and GSK3β-mediated phosphorylation of dynamin-1, which dysregulates endocytosis and alters dendritic spine morphology [41,241]. Spatial correlations between calpain activation, hippocampal neuronal loss, and cognitive impairment are seen in TBI [242], while inhibition of calpain restores LTP and CREB activity, preserving memory-associated transcription [243,244].

CaMKII dysregulation shows region-specific expression changes. Although its upregulation is spatially associated with Aβ plaques [245], transcriptomic studies reveal significant synaptic downregulation in both CTE and AD [246]. Reduced CaMKII activity limits GluA1 trafficking and activity, leading to maladaptive plasticity and cognitive decline [247]. Conversely, pharmacological activation enhances CREB activation, reduces synaptic damage, and improves memory performance [248].

GSK3β overactivity further impairs synaptic plasticity [249], by reducing presynaptic glutamate release, disrupting vesicle recycling, suppressing NMDAR expression, and promoting Aβ-dependent dendritic spine loss, possibly through downregulation of CREB target genes [250,251]. CRMP2, which normally regulates neuronal growth and cytoskeletal organization [252], is inactivated by GSK3β or CDK5, leading to dendritic spine simplification and cognitive deficits [253]. GSK3β-mediated phosphorylation of kinesin light chains also reduces motor motility and disrupts vesicle delivery required for synaptic function [254]. CDK5/p25 activity correlates with tau phosphorylation at Thr217, which is strongly linked to synaptic protein loss, disrupted synaptic and axonal integrity, and cognitive decline, whereas its inhibition reduces these deficits [255].

The PKC family is closely associated with memory formation [50]. PKC activators (e.g., bryostatin1) protect against cognitive deficits in TBI models, possibly by upregulating ADAM10 and downregulating BACE1, thereby reducing Aβ accumulation [256]. In contrast, PKCα gain-of-function mutations in AD enhance catalytic activity and are linked to reduced spine density and impaired cognition [257].

CaN is likewise dysregulated in TBI and AD. Pathological activation by NMDARs and Aβ oligomers promotes NFAT-dependent signaling and AMPAR internalization, weakening synaptic transmission and structural integrity [258,259]. Both tau and Aβ depress CREB activity through CaN-dependent and independent mechanisms, further disrupting memory-related transcription and LTP [260,261]. Inhibition of CaN (e.g., FK506) prevents dendritic spine loss, cortical injury, and AD-related pathology in rodent TBI and AD models [24,262]. Because CaN plays a central role in synaptic depression [69], its blockage also reverses LTP deficits and improves cognition in transgenic mice, rendering it a promising therapeutic or preventive target [263].

In both TBI and AD, calcium dysregulation activates kinases, phosphatases, and proteases that collectively disrupt synaptic architecture, receptor density, axonal transport, and activity-dependent gene expression, leading to LTP failure and cognitive decline.

## 7. Axonal Degeneration

Axonal degeneration represents an early and prominent finding in TBI and AD, initiated by calcium influx and propagated by downstream proteolytic and kinase cascades [264]. In TBI, mechanical membrane disruption permits rapid calcium entry, triggering a wave of enzymatic pathways that degrade the cytoskeleton, impair axonal transport, and promote progressive fragmentation [265]. In AD, similar calcium-dependent transport defects and axonal swellings occur in proximity to neuritic plaques, pointing to shared upstream mechanisms [266].

Calpain is central to this process by cleaving axonal and myelin-associated structural proteins, including spectrin, neurofilaments, and MAPs [57]. Calpain also generates the characteristic 145-kDa spectrin breakdown product (SBDP145) fragment, a candidate biomarker in both TBI and AD [267]. Blocking calpain or calcium influx using inhibitors or calcium channel blockers reduces axonal degeneration and lesion volume in preclinical TBI models [229,268], although incomplete recovery suggests additional calcium-dependent mechanisms are likely involved [269].

Kinases also contribute to axonal degeneration. Pathological CDK5/p25 drives white matter atrophy with focal tau aggregation [270], whereas selective inhibition partially reverses these effects [271]. Combined inhibition of CDK5 and GSK3β offers greater protection than either alone [272], indicating additive or synergistic effects. GSK3β-mediated phosphorylation of CRMP2 compromises cytoskeletal stabilization [252], whereas calpain-dependent CRMP2 cleavage prevents kinesin binding, further disrupting axonal transport [273]. Kinesin downregulation has been observed in early stages of axonal degeneration and is closely associated with local Aβ plaque formation [274]. CaMKII phosphorylation of CRMP2 also contributes to axonal swellings [275].

Calcium dysregulation activates CaN which exacerbates axonal degeneration, partly through mitochondrial impairment [276]. Mitochondrial dysfunction accelerates axonal loss by opening the mitochondrial permeability transition pore (mPTP), releasing calcium and ROS, and triggering irreversible axonal degeneration [277]. CaN inhibition (e.g., cyclosporin A) preserves mitochondrial function and prevents mPTP opening, thereby limiting axonal damage [277,278]. Mitochondrial energy failure disrupts axonal transport, leading to co-accumulation of APP, BACE1, and presenilin in injured axons, creating a permissive microenvironment for amyloidogenic processing [117]. Aβ peptides persist in damaged white matter adjacent to axonal damage months after TBI [279], correlating with increased presenilin expression [280]. Presenilin inhibition reduces white matter injury and improves cognition in animal models [118]. Hyperphosphorylated tau destabilizes the microtubule network, further disrupting APP axonal transport [281], increasing amyloidogenic processing, and reinforcing amyloid toxicity in a self-reinforcing loop [282,283]. Reducing tau improves trafficking and decreases Aβ toxicity [284].

Together, mitochondrial collapse, axonal transport blockade, protease and kinase activation, and amyloid feedback progressively damage axons, leading to chronic white matter degeneration that contributes to synaptic and cognitive deficits in TBI and AD [285].

## 8. Conclusions

Calcium dysregulation is a common and early upstream event in TBI and AD, initiating a network of calcium-dependent signaling cascades that drive neurodegeneration and promote the transition from acute injury to chronic decline. Experimental and human studies consistently show that calcium disturbances appear within minutes to hours after TBI and persistently impair neuronal and glial function. Although brief calcium-dependent enzymatic activation supports physiological function, sustained overload destabilizes homeostasis, sensitizing neurons to degeneration and providing a convergent pathway linking TBI and AD.

Disrupted calcium homeostasis shifts the equilibrium between kinases and phosphatases, promoting tau hyperphosphorylation and amyloidogenic processing, and setting the stage for amyloid plaque and NFT formation. The selective vulnerability of the hippocampus and cerebral white matter to calcium-driven injury likely underlies some of the long-term cognitive sequelae common to both conditions.

Among these mechanisms, calpain has emerged as a central regulator. Preclinical studies show that inhibiting calcium-dependent enzymes exerts neuroprotective effects, but translation into clinical therapies remains elusive. Clarifying the temporal and mechanistic links between calcium dysregulation in TBI and AD will be critical for developing targeted interventions capable of mitigating trauma-related neurodegeneration.

Future therapeutic strategies should aim for precise, combined inhibition of selected calcium-dependent enzymes to block or modulate pathological signaling, while still preserving normal cellular functions. One approach is to target downstream molecular players at critical branch points in the secondary injury cascade. Large, long-term prospective observational studies spanning decades are needed to more accurately define the epidemiological associations between TBI and dementia risk.

Revisiting the calcium hypothesis is timely, because many current AD therapies (e.g., memantine) act on calcium signaling pathways, whereas tau- or amyloid-directed treatments have yielded limited clinical benefit. Approaches to improve TBI classification and phenotyping, including explicit documentation of loss of consciousness, repetition, and injury severity, are essential for harmonizing clinical and experimental research. In parallel, polygenic-score approaches that aggregate small genetic effects across calcium-regulatory genes, using resources such as PGS-Depot and PGSFusion, could help to stratify TBI survivors at risk of calcium-related neurodegeneration. Together, these strategies may help clarify how calcium dysregulation links TBI to AD and ACD and provide a framework for precision-based interventions to prevent or slow TBI-related neurodegeneration.

## Figures and Tables

**Table 1:** Epidemiological evidence supporting TBI as a risk factor for AD and Dementia.

Study Design	Sample Size	Outcome	Pooled Effect Size	95% CI	TBI Characteristics	Included Articles
Systematic Review & Meta-Analysis [7]	7,634,844	Dementia	RR: 1.66	1.42–1.93	No Restrictions (Mild to Severe)	32
Systematic Review & Meta-Analysis [8]	3,149,740	AD	RR: 1.18	1.11–1.25	Mild TBI	5
Systematic Review & Meta-Analysis [9]	2,820,181 8,684,485	AD Dementia	OR: 1.02 OR: 1.81	0.91–1.15 1.53–2.14	No Restrictions (Mild to Severe)	7 21
Meta-Analysis [10]	3,263,207	AD Dementia	OR: 1.03 OR: 1.93	0.06–16.33 1.47–2.55	No Restrictions (Mild to Severe)	18
Systematic Review & Meta-Analysis [11]	7,100,000	AD Dementia	HR: 1.30 HR: 1.95	0.88–1.91 1.55–2.45	No Restrictions (Severity-Dependent Risk)	13
Systematic Review & Meta-Analysis [12]	2,013,197	AD Dementia	RR: 1.51 RR: 1.63	1.26–1.80 1.34–1.99	No Restrictions (Any Head Injury or TBI)	32
Umbrella Systematic Review & Meta-Analysis [13]	20,684,373	AD Dementia	OR: 1.18 OR: 1.81	1.11–1.25 1.53–2.14	No Restrictions (Severity-Dependent Risk)	6
Meta-Analysis [5]	29,181 10,331	AD Dementia	OR: 1.40 OR: 1.36	1.02–1.90 0.84–2.19	Mild TBI	57
Systematic Review & Meta-Analysis [14]	2,351,334	Dementia	OR: 1.96	1.70–2.26	Mild TBI	21
Systematic Review & Meta-Analysis [2]	1,936,593 1,773,342	AD Dementia	OR: 1.60 OR: 1.79	1.44–1.77 1.66–1.92	No Restrictions (Any Head Injury or TBI)	107
Meta-Analysis [15]	4,289,548	AD AD	RR: 1.17 RR: 1.30	1.05–1.29 1.01–1.59	Any TBI (Moderate to Severe)	17

Summary of epidemiological studies assessing TBI as a risk factor for Alzheimer’s Disease and all-cause dementia. The table lists systematic reviews and meta-analyses published over the past 10 years that examine TBI-related neurodegeneration. Reported data include total sample size, outcome (AD or Dementia), effect sizes with corresponding 95% confidence intervals, TBI exposure characteristics (e.g., severity), and number of included studies. Many reviews draw on overlapping primary cohorts or case–control studies; therefore, effect estimates are not additive. Abbreviations: CI: Confidence Interval; HR: Hazard Ratio; OR: Odds Ratio; RR: Risk Ratio.

**Table 2:** Network of calcium-regulated enzymes implicated in TBI-induced neurodegeneration.

Enzyme	Calcium- Dependence	Physiological Function	Major Targets	Pathological Consequence	Enzyme Interactions	Refs.
CaMKII	Direct (Calcium/CaM)	Memory Consolidation, Neurotransmitter Release, Synaptic Plasticity	AMPAR, APP, CREB, CRMP2, NF-κB, NMDAR, Tau	AD Hallmark Pathology, Axonal Damage, Synaptic Dysfunction	Calpain CaN CDK5 GSK3β	[36,37]
CDK5	Indirect (Calpain)	Cytoskeletal Dynamics, Neuronal Development, Synaptic Plasticity	APP, BACE1, CRMP2, DRP1, Dynamin-1, Neurofilaments, PSD-95, STAT, Tau	AD Hallmark Pathology, Axonal Damage, Synaptic Dysfunction	Calpain CaMKII ERK GSK3β PP2A	[38,39,40]
DYRK1A	Indirect (Calpain)	Neuronal Development, Synaptic Plasticity, Transcriptional Control	APP, Dynamin-1, NFAT, Presenilin, STAT, Tau	AD Hallmark Pathology, Neuroinflammation, Synaptic Dysfunction	Calpain, CaN, GSK3β.	[41,42]
ERK	Indirect (Upstream via MAPK cascade, Calpain)	Inflammatory Response, Neuronal Survival, Synaptic Plasticity	α-Secretase, DRP1, NF-κB, STAT, Tau	AD Hallmark Pathology, Neuroinflammation, Oxidative Stress	Calpain CDK5 GSK3β	[43,44]
GSK3β	Indirect (Calpain)	Cell Cycle Regulation, Neuronal Development, Synaptic Plasticity	APP, BACE1, CREB, CRMP2, DRP1, Dynamin-1, Kinesin, NFAT, NF-κB, Presenilin, PSD-95, STAT, Tau	AD Hallmark Pathology, Axonal Damage, Synaptic Dysfunction.	Calpain CaMKII CaN CDK5 DYRK1A ERK PKC PP2A	[45,46,47]
PKC	Direct/Indirect (Isoform dependent, Calpain)	Cell Signaling, Memory Formation, Neuronal Survival	AMPAR, APP, CREB, GAP43, NF-κB, NMDAR, STAT, Tau	AD Hallmark Pathology, Protective (e.g., PKCε) vs. Toxic (e.g., PKCδ), Structural Damage, Synaptic Dysfunction	Calpain GSK3β PP2A	[48,49,50]
CaN	Direct (Calcium/CaM, Calpain)	Inflammatory Response, Neuronal Survival, Synaptic Plasticity	AMPAR, BAD, DRP1, Dynamin 1, MAP, NFAT, NF-κB, NMDAR, Tau	AD Hallmark Pathology, Axonal Damage, Synaptic Dysfunction	Calpain CaMKII DYRK1A GSK3β	[51,52]
PP2A	Indirect (Calcium-binding subunits, Calpain)	Major Tau Phosphatase, Neuronal Survival, Signal Transduction	AMPAR, APP, CREB, DRP1, Neurofilaments, NF-κB, NMDAR, Tau	Decreased Activity: AD Hallmark Pathology, Synaptic Dysfunction	Calpain CDK5 GSK3β PKC	[53,54,55]
Calpain	Direct (Calcium)	Apoptosis Regulation, Cytoskeletal Remodeling, Signal Transduction	AMPAR, APP, CRMP2, DRP1, Dynamin-1, GAP43, IP3R, MAP, NCX, Neurofilaments, NMDAR, p35, PMCA, Presenilin, PSD-95, Spectrin, Tau, Tubulin, VGCC	Aβ Pathology, Axonal Damage, Enzyme Modulation, Synaptic Dysfunction, Tau Fragmentation	CaMKII CaN CDK5 DYRK1A ERK GSK3β PKC PP2A	[56,57]

Comprehensive overview of calcium-regulated enzymes implicated in trauma-related neurodegeneration and their pathological roles. The table summarizes calcium-dependence (direct or indirect), physiological functions, major molecular targets, and pathological consequences of calcium-modulated enzymes. It also highlights key enzymatic interactions, emphasizing the interconnected signaling network that collectively drives hallmark neuropathological features. Abbreviations: APP: Amyloid Precursor Protein; CaM: Calmodulin; CaMKII: Calcium/Calmodulin-Dependent Protein Kinase II; CaN: Calcineurin; CDK5: Cyclin-Dependent Kinase 5; CREB: cAMP Response Element-Binding Protein; CRMP2: Collapsin Response Mediator Protein 2; DRP1: Dynamin-Related Protein 1; DYRK1A: Dual-Specificity Tyrosine Phosphorylation-Regulated Kinase 1A; ERK: Extracellular Signal-Regulated Kinase; GAP43: Growth Associated Protein 43; GSK3β: Glycogen Synthase Kinase 3β; MAP: Microtubule Associated Protein; NFAT: Nuclear Factor of Activated T Cells; NF-κB: Nuclear Factor Kappa-B; PKC: Protein Kinase C; PP2A: Protein Phosphatase 2A; PSD-95: Postsynaptic Density Protein 95; Refs: References; STAT: Signal Transducer and Activator of Transcription.

## Data Availability

Not applicable, since no new data were created.

## Abbreviations

AD	Alzheimer’s Disease
ADAM10	A Disintegrin and Metalloproteinase 10
ACD	All-Cause Dementia
AEP	Asparaginyl Endopeptidase
AICD	APP Intracellular Domain
AMPAR	α-Amino-3-Hydroxy 5-Methyl-4-Isoxazolepropionate Receptor
APOE4	Apolipoprotein ε4
APP	Amyloid Precursor Protein
ATP	Adenosine Triphosphate
Aβ	Amyloid-Beta
BACE1	Beta-site Amyloid Precursor Protein Cleaving Enzyme 1
BAD	Bcl-2-Associated Death Promoter
CaM	Calmodulin
CaMKII	Calcium/Calmodulin-Dependent Protein Kinase II
CaN	Calcineurin
CDK5	Cyclin-Dependent Kinase 5
CI	Confidence Interval
CICR	Calcium-Induced Calcium Release
CP-AMPAR	Calcium-Permeable AMPAR
CREB	cAMP Response Element-Binding Protein
CRMP2	Collapsin Response Mediator Protein 2
CSF	Cerebrospinal Fluid
CTE	Chronic Traumatic Encephalopathy
DRP1	Dynamin-Related Protein 1
DYRK1A	Dual-specificity tyrosine phosphorylation-regulated kinase 1A
eIF2α	Eukaryotic Initiation Factor 2α
ER	Endoplasmic Reticulum
ERK	Extracellular Signal-Regulated Kinase
FDA	Food and Drug Administration
FOXP2	Forkhead Box P2
FTO	Fat Mass and Obesity-Associated
GAP43	Growth-Associated Protein 43
GSK3β	Glycogen Synthase Kinase 3β
GWAS	Genome-Wide Association Study
HR	Hazard Ratio
IP3R	Inositol 1,4,5-Trisphosphate Receptor
LTP	Long-Term Potentiation
MAM	Mitochondria-Associated ER Membrane
MAP	Microtubule-Associated Protein
MAPT	Microtubule-Associated Protein Tau
MAPK	Mitogen-Activated Protein Kinase
MCU	Mitochondrial Calcium Uniporter
mGluR	Metabotropic Glutamate Receptor
mNCX	Mitochondrial Sodium/Calcium Exchangers
mPTP	Mitochondrial Permeability Transition Pore
NCAM1	Neural Cell Adhesion Molecule 1
NCX	Sodium/Calcium Exchangers
NFAT	Nuclear Factor of Activated T Cells
NFT	Neurofibrillary Tangles
NF-κB	Nuclear Factor Kappa-B
NMDAR	N-Methyl-D-Aspartate Receptor
OR	Odds Ratio
PHF	Paired Helical Filament
PKB	Protein Kinase B
PKC	Protein Kinase C
PMCA	Plasma Membrane Calcium-ATPase
PP2A	Protein Phosphatase 2A
PSD-95	Postsynaptic Density Protein 95
Refs.	References
ROS	Reactive Oxygen Species
RR	Risk Ratio
RyR	Ryanodine Receptor
SBDP145	145-kDa Spectrin Breakdown Product
SERCA	Sarcoplasmic–Endoplasmic Reticulum Calcium-ATPase
SNP	Single-Nucleotide Polymorphism
SOC	Store-Operated Calcium Channel
STAT	Signal Transducer and Activator of Transcription
TBI	Traumatic Brain Injury
TRP	Transient Receptor Potential Channel
UPR	Unfolded Protein Response
VGCC	Voltage-Gated Calcium Channels
